# The health status of South African youth joining Youth Employment Initiatives: a health promotion opportunity

**DOI:** 10.1093/heapro/daaf061

**Published:** 2025-05-22

**Authors:** Mimi Mhlaba, Tsholofelo Mohitshane, Nqobile Mnisi, Elelwani Nevhufumba, Georgia Torres, Florian Zech, Carey Jooste, Bridget Vermeulen, Lisa J Ware

**Affiliations:** SAMRC/Wits Developmental Pathways for Health Research Unit, Faculty of Health Sciences, School of Clinical Medicine, University of the Witwatersrand, Chris Hani Baragwanath Hospital, 26 Chris Hani Road, Soweto 1864, (Egoli) Johannesburg, South Africa; SAMRC/Wits Developmental Pathways for Health Research Unit, Faculty of Health Sciences, School of Clinical Medicine, University of the Witwatersrand, Chris Hani Baragwanath Hospital, 26 Chris Hani Road, Soweto 1864, (Egoli) Johannesburg, South Africa; SAMRC/Wits Developmental Pathways for Health Research Unit, Faculty of Health Sciences, School of Clinical Medicine, University of the Witwatersrand, Chris Hani Baragwanath Hospital, 26 Chris Hani Road, Soweto 1864, (Egoli) Johannesburg, South Africa; Department of Exercise Science and Sports Medicine, University of the Witwatersrand Education Campus, 27 St Andrews Road, Parktown 2193, (Egoli) Johannesburg, South Africa; Department of Exercise Science and Sports Medicine, University of the Witwatersrand Education Campus, 27 St Andrews Road, Parktown 2193, (Egoli) Johannesburg, South Africa; Founder and Managing Director, AMANDLA Social Enterprises, 16 Baker Street, 7th Floor, Rosebank 2196, (Egoli) Johannesburg, South Africa; Head of Innovation, Development Bank of Southern Africa (DBSA), 1258 Lever Road, Headway Hill, Midrand 1685, South Africa; SAMRC/Wits Developmental Pathways for Health Research Unit, Faculty of Health Sciences, School of Clinical Medicine, University of the Witwatersrand, Chris Hani Baragwanath Hospital, 26 Chris Hani Road, Soweto 1864, (Egoli) Johannesburg, South Africa; SAMRC/Wits Developmental Pathways for Health Research Unit, Faculty of Health Sciences, School of Clinical Medicine, University of the Witwatersrand, Chris Hani Baragwanath Hospital, 26 Chris Hani Road, Soweto 1864, (Egoli) Johannesburg, South Africa

**Keywords:** health, mental health, NEET youth, South Africa, Youth Employment Initiatives, young adults

## Abstract

Youth Employment Initiatives (YEIs) support young adults to enhance their employability but infrequently consider their health. This study assessed the physical and mental health of South African youth starting YEIs to understand the need for integrated health service components. Youth (18–35 years, *n* = 193, 76% female, mean age 24.6 ± 3.6 years) were recruited using a purposive sampling strategy from the YEI programme within South Africa. Sociodemographic and health data were collected by survey, with a subsample (*n* = 90) undergoing cardiometabolic assessment. One-third of youth reported thoughts of suicide or self-harm in the past 2 weeks, with significantly higher levels of depression and anxiety in youth under 25 years. Obesity levels and blood pressure were significantly higher in youth 25–35 years (68% overweight/obese, 26% hypertensive). Our findings support the urgent need for integrated physical and mental health support within youth programmes to reduce both the future and current burden of non-communicable diseases.

Contribution to Health Promotion

- In many African countries, youth unemployment is a significant risk factor for chronic disease.
- We present the health needs of African youth joining YEIs to inform programmatic change.
- The findings provide critical data to design and contribute to targeted health promotion interventions tailored to youth health needs.
- The study also promotes a comprehensive approach to youth health, incorporating physical, mental and social aspects in health promotion efforts.
- By identifying unmet health needs, this research supports the development of policies that improve access to essential health services for youth, facilitating resource allocation for effective health promotion programmes.

## BACKGROUND

The increasing trend of youth not in employment, education, or training (NEET) is a major concern, with estimates suggesting that between one-third to a half of youth aged 15–34 years in South Africa are NEET (*Quarterly Labour Force Survey. Quarter 1: 2024* 2024). This high proportion of NEET youth impacts the economy through decreased productivity and tax revenue, and increased spending on social services, as well as impacting the livelihoods and health of the NEET youth themselves (Graham *et al*. 2019, Mngoma and Ayonrinde 2023). Previous research has shown that NEET youth are among the most vulnerable groups in society, with extended periods of disconnection from the labour market and education or training opportunities, thereby increasing their risk of remaining trapped in income poverty, and their vulnerability to poor mental and physical ill health (Matli and Ngoepe 2021a).

The impact of being NEET, especially over extended periods, is also associated with poor health behaviours such as low physical activity levels and increased substance use (tobacco, alcohol, and drugs) (Stewart *et al*. 2017, Gutiérrez-García *et al*. 2018, Tanton *et al*. 2021, Gariépy *et al*. 2022). With efforts to secure employment and training opportunities, NEET youth often struggle to prioritize their health, frequently leading to limited focus on health risks (Ware *et al*. 2019) and increasing the likelihood of acute and long-term health complications. Additionally, compared to their peers who are engaged in education or employment, NEET youth often face dissatisfaction, low self-esteem, frustration, and hopelessness, leading to a cycle of social drift and economic inactivity (Matli and Ngoepe 2021b). This is exacerbated by fear of social marginalization and long-term economic disadvantage and may further limit prospects for a career and re-engaging in the labour market (Rodwell *et al*. 2018).

In response to the NEET crisis, there have been multiple programmes to facilitate entry to the workforce through enhancing skills development and providing work experience to NEET youth in South Africa, including the Presidential Youth Employment Initiative (PYEI) launched in 2020 (Dicks 2020). Such programmes saw over 320 000 youth appointed as education assistants to support teachers and help children catch up with learning that was missed during the COVID-19 pandemic school shutdowns (Department of Basic Education 2025). The impact of such employment initiatives on long-term employment is unclear, with critics promoting the need for enhanced economic impact through more public-private partnerships, consideration of labour market demands, and overall better alignment between supply and demand factors (Marock *et al*. 2023, Sibanda 2023). However, as these programmes are typically delivered over 6-12 months, they also present a valuable opportunity for engaging with NEET youth to optimize physical and mental health to ensure maximal benefit.

Research in the United Kingdom showed that youth participating in Youth Opportunity Schemes reported lower levels of depression compared to unemployed youth (Branthwaite andand Garcia 1985), highlighting the potential mental health benefits of such programmes, beyond reducing short-term unemployment (Mawn *et al*. 2017). Indeed initiatives informed by labour market policies in several countries foster integrated health, education, and job training for youth development. For example, the employment initiative in the European Union aims to enhance access to job opportunities for young seekers while prioritizing mental health assistance (Hall *et al*. 2015). In Bulgaria, the ‘Activate Inactive Persons’ National Programme implemented by the National Employment Agency in 2008, assists economically inactive and unemployed youth by offering psychological support, and motivation training, and supports youth with re-entry into the workforce (Hall *et al*. 2015). These initiatives emphasize mental health integration and social support from YEIs may play a significant role in mitigating stress and helping NEET youth cope with life challenges. However, within the South African and broader African context, the physical health of NEET youth may also be critical as previous research shows one in every five children under 18 years in the region already have increased blood pressure (Crouch *et al*. 2022). Understanding the health needs and the burden of poor health in NEET youth joining youth employment initiatives (YEIs) could offer valuable insights and guide future policy efforts to address South Africa’s NEET youth crisis.

Therefore, the objective of the study was to evaluate the physical and mental health status of NEET youth as they start YEIs to inform the need for integrated services within these programmes. A secondary objective was to collect data to provide a baseline for the future assessment of the impact of current YEIs on the health and well-being of youth.

## METHODS

### Participants and study setting

Previously NEET youth (18–35 years) were recruited using a purposive sampling strategy between October 2020 and December 2023. Recruitment adverts were circulated to youth through agencies managing YEI programmes. Purposive sampling of YEI programmes was undertaken using a snowball approach to recruit more programmes. Random sampling across YEI programmes in the province was not undertaken as currently there appears no central register for such programmes and the duration, intensity, and cohort size of various programmes can differ considerably. Voluntary participation in the study was reinforced by the study team to potential youth participants. All youth had been recruited to the YEI programmes through advertising and interviews. To be eligible for the study, the youth had to be within the first 8 weeks of their enrolment in a YEI programme, and the programme had to be at least 6 months in duration. The subsample with cardiometabolic testing consisted of a local YEI affiliated with the University such that University staff could conduct the additional assessments through the available University staff exercise programmes. All YEI programmes sampled were located within the Gauteng Province, the most populated province of South Africa with the highest number of working age youth, the largest share of discouraged youth work-seekers, and a youth unemployment rate in 2024 of 49% (Marginalised Groups Series VII: The Social Profile of the Youth/Statistics South Africa 2025). Many included programmes were located within the large township of Soweto: an area of historical deprivation with a population of around 1.7 million.

### Ethical considerations

The study was approved by the Human Research Ethics Committee (Medical) of the University of the Witwatersrand [M200941]. All participants gave written informed consent prior to data collection.

### Data collection

Sociodemographic, health, and health behaviour data were collected using a self-administered online survey on REDCap (Harris *et al*. 2019). All participants completed the survey at their respective workplaces with support from the study team either in person or through remote videoconferencing. Any participants reporting moderate or severe levels of depression, anxiety, or any thoughts of suicide or self-harm, or with a blood pressure reading indicating potential hypertension were contacted by a professional nurse and referred to appropriate health services for further evaluation.

#### Food security and substance use

Food security was assessed using an adapted Community Childhood Hunger Identification Project (CCHIP) (Wehler *et al*. 1992). Questions asked included: (i) ‘Does your household ever run out of money to buy food?’; (ii) ‘Do you ever cut the size of meals or skip them because there isn’t enough money or food?’; (iii) ‘Do you go to bed hungry because there isn’t enough money to buy food?’. Each question was followed by two questions: (i) ‘Has it happened in the past 30 days?’; (ii) ‘Has it happened at least 5 times in the last 30 days?’. Each positive response was scored 1 (negative scored as zero), providing a food security score ranging from 0 to 9. Scores in the ranges of 0–1, 2–4, and 5–9 indicated food security, at risk of food insecurity, and food insecurity, respectively. Previous and current cigarette smoking and other tobacco use were assessed using questions from the Global Adult Tobacco Survey (GATS) (Palipudi *et al*. 2016). The World Health Organization 10-item Alcohol Use Disorders Identification Test (AUDIT) (Saunders *et al*. 1993) was used to assess alcohol consumption and non-prescribed drug use was assessed with the question: ‘Have you ever taken non-prescribed drugs?’ followed by a question to determine if this was recently (in the last 12 months).

#### Psychosocial measures

The Perceived Stress Scale (PSS-10) (Cohen *et al*. 1983) was used to assess self-reported current stress levels, and the Generalized Anxiety Disorder 7 (GAD-7) questionnaire was used to evaluate the presence and severity of generalized anxiety disorder (Spitzer *et al*. 2006). The Patient Health Questionnaire (PHQ-9) was used to assess the possible presence and severity of depression symptoms (Kroenke *et al*. 2001) with suicidal ideation or self-harm assessed by the response to the PHQ-9 question 9 ‘Over the last 2 weeks, how often have you been bothered by thoughts that you would be better off dead or of hurting yourself in some way?’.

#### Cardiometabolic measures

In a subsample of youth participants based in Soweto, cardiometabolic assessments were conducted. Height was measured with a stadiometer and weight with a calibrated digital scale [Seca GmbH, Hamburg, Germany]. Waist circumference was measured at the midway point between the iliac crest and the lowest rib by trained research assistants. Body mass index (BMI kg/m^2^) and waist-to-height ratio (waist and height measured in centimetres) were calculated by applying accepted thresholds (Ashwell and Gibson 2014, *World Health Organization* 2024) to determine healthy and less healthy body habitus. Blood pressure was measured with a validated automated device [M3 Omron, Japan] and an appropriate size cuff on the right arm with the participant seated at rest for at least five minutes prior to the measurement, legs uncrossed, and right arm resting at the level of the heart. Hypertension status was determined by systolic and diastolic readings and the use of any antihypertensive medication.

### Data analysis

Data were analyzed using SPSS version 29.0.2.0 [IBM, New York] for all participants and compared between two age groups (18–24.9 years and 25–35 years) to facilitate the design of targeted interventions. Continuous data were checked using visual inspection of Q–Q plots, with data deviating from a normal distribution log-transformed for analysis. Age group comparisons were performed using *T*-tests for continuous variables or chi-square tests for categorical variables, with likelihood ratio chi-square tests used where cell frequencies were <5 and combining categories was not possible.

## Results

The study included 193 youth (76% female, 97% black) with a mean age of 24.6 ± 3.6 years (**Table 1**). Most participants (86%) had a high school certificate with half (51%) having attended some tertiary education, although, in both age groups, only around half of these had completed and graduated from tertiary education. Across the age groups, 1 in 10 youth (11%) lived in informal housing (a shack). While there were no differences between younger (18–24y) and older (25–35y) participants in relationship status with most reporting being single (62%), twice as many of the older group reported having one or more children (59%) compared to the younger group (28%; *P* < 0.001).

**Table 1.** Sociodemographic characteristics of youth joining Youth Employment Initiatives in Gauteng Province by age group (*n* = 193).

	Total (*n* = 193)	18–24 years (*n* = 93)	25-35 years (*n* = 100)	*P*-value
Age, years	24.6 ± 3.6	21.6 ± 1.8	27.5 ± 2.2	*N/A*
Female, *n* (%)	146 (76)	71 (76)	75 (75)	0.828
Ethnicity, *n* (%)				
Black African	187 (97)	88 (95)	99 (99)	0.080
Mixed race	6 (3)	5 (5)	1 (1)	
First language, *n* (%)				
IsiZulu	75 (39)	35 (38)	40 (40)	0.667
SeSotho	34 (17)	13 (14)	21 (21)	
SeTswana	23 (12)	14 (15)	9 (9)	
IsiXhosa	21 (11)	11 (12)	10 (10)	
Northern SeSotho	13 (7)	7 (7)	6 (6)	
Other	27 (14)	13 (14)	14 (14)	
Relationship status, *n* (%)				
Single	120 (62)	65 (70)	55 (55)	0.186
Married/in relationship (cohabiting)	47 (24)	19 (20)	28 (28)	
Married/in relationship (not cohabiting)	23 (12)	8 (9)	15 (15)	
Refused to answer	3 (2)	1 (1)	2 (2)	
Has a child or children, n (%)	84 (44)	26 (28)	58 (59)	**<0.001**
Education, *n* (%)				
Has High School Certificate	165 (86)	78 (84)	87 (87)	0.376
Attended higher education	98 (51)	37 (40)	61 (61)	**0.007**
Graduated higher education	55 (56)	19 (51)	36 (59)	0.313
Number of previous jobs, *n* (%)				
None	45 (23)	36 (39)	9 (9)	**<0.001**
One to three	133 (69)	55 (59)	78 (78)	
More than three	15 (8)	2 (2)	13 (13)	
Housing type, *n* (%)				
Brick/stone/concrete building	171 (87)	83 (89)	88 (88)	0.785
Informal shack	22 (11)	10 (11)	12 (12)	

Values are number (*n*) and percentage (%) of participants or mean ± standard deviation. Comparisons between age groups are conducted with chi-square tests or likelihood ratio chi-square if cell count < 5 for categorical data, and by Students *t*-test for continuous data. Bold values denote statistical significance (*P* < 0.050).

Overall, one-third of the group reported being food insecure or at risk of food insecurity (**Table 2**). While most participants (87%) either abstained from alcohol or engaged in low-risk alcohol consumption, 13% of the sample reported potentially hazardous alcohol use behaviour including the risk of alcohol dependence, with no statistically significant differences between the older and younger age groups. Current cigarette smoking was reported by 11% of participants, with no difference by age group. In contrast, twice as many of the younger group (32%) reported recent use of non-cigarette tobacco products when compared to the older group (16%, *P* = 0.036). This was also observed for recent non-prescription drug use (16% of the younger group versus 7% of the older group), though this failed to reach statistical significance (*P* = 0.064).

**Table 2.** Nutrition and health behaviours of youth joining Youth Employment Initiatives in Gauteng Province by age group (*n* = 193).

	All (*n* = 193)	18–24 years (*n* = 93)	25–35 years (*n* = 100)	*P*-value
Food security status, *n* (%)				
Food secure	135 (70)	64 (69)	71 (71)	0.912
At risk of food insecurity	37 (19)	19 (20)	18 (18)	
Food insecure	21 (11)	10 (11)	11 (11)	
Alcohol use, *n* (%)				
Abstainer	54 (28)	25 (27)	29 (29)	0.946
Low risk use	113 (59)	55 (59)	58 (58)	
Hazardous or harmful use	21 (11)	11 (12)	10 (10)	
Likelihood of dependence	5 (2)	2 (2)	3 (3)	
Smoking, *n* (%)				
Never smoked	142 (74)	75 (80)	67 (67)	0.131
Previously smoker	29 (15)	10 (11)	19 (19)	
Current smoker	21 (11)	8 (9)	13 (13)	
Refused to answer	0	1 (1)	1 (1)	
Other tobacco use, *n* (%)				
Never	135 (70)	56 (60)	79 (79)	**0.036**
Not recently (> 1 year ago)	8 (4)	5 (6)	3 (3)	
Within past 12 months	46 (24)	30 (32)	16 (16)	
Refused to answer	4 (2)	2 (2)	2 (2)	
Recreational drug use, *n* (%)				
Never	158 (82)	69 (74)	89 (89)	0.064
Not recently (> 1 year ago)	10 (5)	7 (8)	3 (3)	
Within past 12 months	22 (11)	15 (16)	7 (7)	
Refused to answer/don’t know	3 (2)	2 (2)	1 (1)	
Current/previous drug users who feel they ought to cut down on drug use	19 (59)	16 (68)	4 (40)	—

Values are number (*n*) and percentage (%) of participants. Comparisons between age groups are conducted with chi-square tests or likelihood ratio chi-square if cell count <5 for categorical data, and by students *T*-test for continuous data. Bold values denote statistical significance (*P* < 0.050).

Most youth (78%) reported either moderate or high current perceived stress levels (**Table 3**), with no difference by age group. However, both the anxiety and depression scores were significantly higher in the younger age group. The younger group more frequently reported symptoms of moderate (22% vs. 16% in the older group) or severe anxiety (9% versus 6% in the older group, *P* = 0.010) and moderate to severe depression (42% versus 26% in the older group, *P* = 0.016). One-third of youth reported considering self-harm or suicide in the past 2 weeks with no statistically significant difference by age group.

**Table 3.** Stress and mental health status of youth joining Youth Employment Initiatives in Gauteng Province by age group (*n* = 193).

	All (*n* = 193)	18–24 years (*n* = 93)	25–35 years (*n* = 100)	*P*-value
Perceived stress score	18.3 ± 6.4	19.1 ± 6.5	17.6 ± 6.2	0.116
Low-perceived stress, n (%)	43 (22)	18 (19)	25 (25)	0.500
Moderate-perceived stress, n (%)	129 (67)	63 (68)	66 (66)	
High-perceived stress, n (%)	21 (11)	12 (13)	9 (9)	
Anxiety score	6.2 ± 5.5	7.2 ± 5.3	5.3 ± 5.4	**0.014**
Minimal anxiety, n (%)	90 (47)	32 (34)	58 (58)	**0.010**
Mild anxiety, n (%)	53 (28)	33 (35)	20 (20)	
Moderate anxiety, n (%)	36 (19)	20 (22)	16 (16)	
Severe anxiety, n (%)	14 (7)	8 (9)	6 (6)	
Depression score	7.7 ± 6.4	8.7 ± 6.3	6.7 ± 6.3	**0.029**
None or minimal, n (%)	72 (37)	26 (28)	46 (46)	**0.016**
Mild, n (%)	56 (29)	28 (30)	28 (28)	
Moderate, n (%)	40 (21)	23 (25)	17 (17)	
Moderately severe, n (%)	12 (6)	10 (11)	2 (2)	
Severe, n (%)	13 (7)	6 (6)	7 (7)	
Thoughts of suicide/self-harm				
Not at all, n (%)	135 (70)	60 (64)	75 (75)	0.262
Several days, n (%)	30 (15)	15 (16)	15 (15)	
More than half the days, n (%)	13 (7)	9 (10)	4 (4)	
Nearly every day, n (%)	15 (8)	9 (10)	6 (6)	

Values are number (*n*) and percentage (%) of participants or mean ± standard deviation. Comparisons between age groups are conducted with chi-square tests or likelihood ratio chi-square if cell count <5 for categorical data, and by Students *t*-test for continuous data. Bold values denote statistical significance (*P* < 0.050). Anxiety and depression scores were reported for the last two weeks. Perceived stress score was reported for the last month.

In the subsample with cardiometabolic measures (**Table 4**), the levels of overweight and obesity were significantly higher in the older compared to the younger group by all measures used (waist circumference, BMI, and waist-to-height ratio). One-third (31%) of the younger group had a BMI indicative of overweight or obesity, and this was more than doubled in the older group (68%, *P* = 0.004). The older group also showed significantly higher systolic and diastolic blood pressures, with 26% categorized as hypertensive.

**Table 4.** Physical health status of youth joining Youth Employment Initiatives in Gauteng Province by age group (subgroup analysis, *n* = 90).

	All (*n* = 90)	18–24 years (*n* = 36)	25–35 years (*n* = 54)	*P*-value
Age (years)	24.8 ± 3.0	21.7 ± 1.6	26.9 ± 1.5	*N/A*
Female, *n* (%)	70 (78)	30 (83)	40 (74)	0.301
Height (cm)	162 ± 9	161 ± 9	163 ± 9	0.443
Weight (kg)	70.1 ± 18.6	61.5 ± 14.3	75.9 ± 19.0	**<0.001**
Waist circumference (cm)	83.0 ± 16.4	75.6 ± 13.7	88.0 ± 16.2	-
Log waist circumference	4.4 ± 0.2	4.3 ± 0.2	4.5 ± 0.2	**<0.001**
Body mass index (kg/m^2^)	26.7 ± 7.3	23.6 ± 5.8	28.7 ± 7.6	-
* Underweight, n (%)*	4 (4)	2 (5)	2 (4)	**0.004**
* Healthy weight, n (%)*	38 (42)	23 (64)	15 (28)	
* Overweight, n (%)*	25 (28)	5 (14)	20 (37)	
* Obese, n (%)*	23 (26)	6 (17)	17 (31)	
Log body mass index	3.3 ± 0.3	3.1 ± 0.2	3.3 ± 0.3	**<0.001**
Waist-to-height ratio	0.51 ± 0.11	0.47 ± 0.09	0.54 ± 0.10	–
* Healthy (< 0.5), n (%)*	43 (48)	23 (64)	20 (37)	**0.012**
* Overweight (≥ 0.5), n (%)*	47 (52)	13 (36)	34 (63)	
Log waist-to-height ratio	-0.69 ± 0.19	-0.77 ± 0.17	−0.63 ± 0.18	**<0.001**
Systolic blood pressure (mmHg)	118 ± 14	113 ± 14	122 ± 14	**0.001**
Diastolic blood pressure (mmHg)	80 ± 11	76 ± 10	83 ± 11	**0.002**
Hypertension status, *n (%)*				
* Normotensive (< 120/80)*	38 (42)	19 (53)	19 (35)	0.077
* Pre-hypertensive (120-139/80-89)*	35 (39)	14 (39)	21 (37)	
* Hypertensive (≥ 140/90) or treated to target*	17 (19)	3 (8)	14 (26)	

Values are number (*n*) and percentage (%) of participants or mean ± standard deviation. Comparisons between age groups are conducted with chi-square tests or likelihood ratio chi-square if cell count <5 for categorical data, and by *t*-test for continuous data. Bold values denote statistical significance (*P* < .050).

## DISCUSSION

This study aimed to characterize the health status of previously NEET youth joining YEIs to provide a foundation for assessing the impact of YEIs on youth health and to evaluate the need to embed health services within these programmes. Our findings showed that many young people joining YEIs are at immediate risk for depression, and anxiety and have recently considered self-harm, especially in the younger age range of 18–24 years who also reported more tobacco and recreational drug use. The findings also showed that overweight and obesity, and elevated blood pressure are common and increased with age in this young adult group with increased risk for cardiometabolic disease including diabetes, heart disease, and stroke.

Evidence suggests that unemployment among NEET youth has acute adverse effects on health, such as increased rates of poor mental well-being, depression, and suicidal behaviours (Fergusson *et al*. 2001, Siegrist *et al*. 2009, Stewart *et al*. 2017). Our results showed high numbers of youth with increased levels of perceived stress, and moderate to severe anxiety levels, suggesting that stress and anxiety are a prevalent issue among South African NEET youth. Our results are consistent with other research that showed high levels of stress and anxiety among NEET youth (Gariépy *et al*. 2022). Arguably, NEET youths’ heightened stress levels may stem from the assumption that they face financial uncertainty and hence find it difficult to meet their basic needs (Rahmani and Groot 2023). Moreover, higher stress levels in NEET youth may also be a result of the absence of structure and routine associated with unemployment (Rahmani and Groot 2023). However, our data is from previously NEET youth who have started YEIs, which suggests that engaging with these programmes, including receiving the financial stipend, at least in the first few weeks, may be insufficient to alleviate this and additional interventions are needed.

Our study also indicated that many NEET youth joining YEIs may be experiencing moderate to severe symptoms of depression. Depression symptoms observed in NEET youth have been previously attributed to their social isolation when disengaged from social settings such as school and the workplace (Jongbloed and Giret 2022). However, the youth in our study had already connected with their trainee peers in a workplace setting. This again suggests that additional interventions are needed to support improvements in youth mental health and well-being. Worryingly, we observed one-third of youth expressing recent suicidal ideation or self-harm, further highlighting the urgency of such interventions.

Research from NEET youth in Mexico City also reported an increased risk of incident suicidal behaviours (Gutiérrez-García *et al*. 2017). In response to these risks, Canadian NEET youth (14–29 years) expressed a strong preference for employment initiatives that integrate mental health and wellness in addition to skill-building, life skills training, and long-term pathways to education and employment (Quinlan-Davidson *et al*. 2024). Such interventions may significantly improve youth mental health by providing comprehensive support and long-term economic security, thus potentially reducing stress.

The association between unemployment and poor mental and physical health may in part be exacerbated by poor health behaviours such as poor diet, smoking, and alcohol use (Voss *et al*. 2004, Stewart *et al*. 2017). Prior research has suggested that NEET youth are more likely to smoke (Hagquist and Starrin 1996, Baggio *et al*. 2015, [Bibr CIT0019][Bibr CIT0021]) and increase alcohol use or abuse/dependence (Hagquist and Starrin 1996, [Bibr CIT0014][Bibr CIT0021]), although these findings are not consistently observed (Hammer 1992, Stewart *et al*. 2017). Indeed, our study showed that most participants abstained from smoking and engaged in no or low-risk alcohol consumption. However, around one in ten youth did report smoking and/or potentially hazardous alcohol use behaviour, possibly as a coping mechanism to deal with stress (Stewart *et al*. 2017), suggesting the need for targeted support within YEIs. We did observe that more of the younger group reported using non-cigarette tobacco products and non-prescription drugs. Training older youth to offer peer-led counselling for younger age groups and engaging younger groups in individual or group therapy programmes for addiction-related challenges could be beneficial. Peer-led interventions have shown success in reducing the use of tobacco, alcohol, and cannabis among adolescents (MacArthur *et al*. 2016) and may be cost-effective within a low-resource setting, while also contributing to skills development among youth groups.

Our data suggested that, at least in the initial stages of taking part in a YEI, significant levels of food insecurity remain in a third of youth. Food security is deeply intertwined with the broader social determinants of health, such as access to resources, and systemic inequalities (Azevedo *et al*. 2023). Additionally, with significant numbers of youth at risk of food insecurity, our results further indicate that any health behaviour interventions should consider access to healthy foods. For example, interventions that offer food subsidies, discounts, or cash incentives on healthy foods for youth in YEIs may offer the double benefit of improving access to healthy food options and reducing food insecurity.

Many of South Africa’s NEET youth only have matric (high school) education or less and a lower education level increases the risk of being NEET, while a tertiary education significantly improves an individual’s chances of successfully entering the labour market (Graham and Mlatsheni 2015, [Bibr CIT0011][Bibr CIT0017]). While half of the youth in our study had attended a tertiary education institution, only half of these again could complete and graduate. Investing in adult basic education and services at YEIs to support pipelines into (or back into) tertiary education would further positively impact youth employment outcomes (Anowor *et al*. 2023). This would also potentially improve health literacy levels and decision-making around health behaviours (Darmon and Drewnowski 2008, Stewart *et al*. 2017), and the ability to participate in health-promoting activities such as planning nutrient-dense meals (Borraccino *et al*. 2016, Stewart *et al*. 2017).

Furthermore, despite the high demand for skilled labour, persistent skills mismatch in both technical and non-technical (soft) skills among youth contributes to continuing high levels of unemployment (Habiyaremye *et al*. 2022). Even when youth enter the workforce, working poverty in South Africa remains a significant problem. South Africa remains a country with persistently high levels of poverty and inequality, where workers are not only paid wages below the amount necessary to maintain decent living standards but legally employees are not entitled to health or retirement benefits (Feder and Yu 2020).

Our findings agree with previous research that showed high levels of obesity in NEET youth (ten times higher compared to their non-NEET peers) (Stewart *et al*. 2017, Höld *et al*. 2018). Additionally, the average BMI of individuals with a lower socioeconomic status (SES) tends to remain higher compared to those with a higher SES over the life course (Newton *et al*. 2017), suggesting that intervening in early adulthood may have lifetime consequences for health. This shows the importance of health-promoting behavioural interventions from a young age to allow individuals to maintain their health as they age (Höld *et al*. 2018). Data on the prevalence of hypertension in NEET youth specifically is scarce, but our results indicated that prehypertension and hypertension levels are concerning in this group and that not intervening in early adulthood leads to significant levels of hypertension by the time youth reach their late 20s. This may be further exacerbated by the increasing levels of overweight and obesity in this group, a global problem that contributes to increased blood pressure and hypertension (Jia *et al*. 2024). The younger group showed lower levels of overweight and obesity and lower blood pressure, suggesting that primordial prevention efforts in this group may be highly effective. Interventions focused on youth cardiovascular health are warranted, especially within the context of South Africa’s growing burden of non-communicable disease morbidity and mortality and other efforts at curbing this for the most part failing (Nyaaba *et al*. 2017).

Lastly, our participants were all of reproductive age and predominantly black females, the group most likely to be affected by NEET status (De Lannoy and Mudiriza 2019). As such, integrated health interventions in this population may serve triple dividends in (i) removing barriers (such as poor mental health) for optimal engagement with YEI training programmes, (ii) promoting longer-term economic and health benefits for youth, and (iii) promoting improved maternal, paternal and child health for subsequent generations.

### Study strengths and limitations

The study employed a comprehensive approach to understanding the health status of NEET youth joining YEIs, which allowed for the consideration of a wide range of factors that could potentially influence youth health and well-being and engagement with YEI programmes. Moreover, NEET youth were recruited from YEI programmes located in the South African province with the highest prevalence of NEET youth. One limitation is the absence of a comparison group of non-NEET youth. However, the objective was to inform the need for programmatic changes to current YEIs, which are not typically accessed by non-NEET youth. A further limitation is the exclusion of NEET youth not currently enrolled in YEIs. However, our findings do appear consistent with many global studies showing that the NEET population report worse health status across multiple domains compared to their non-NEET peers (Chandler and Lozada 2021).

Further research is necessary to understand the impact of compromised physical and mental health on youths’ participation in Youth Employment Initiatives. Additionally, it is essential to investigate how these programs, in turn, affect the overall health and well-being of their participants.

## CONCLUSION

Being unemployed and not in education appears to have profound impacts on youth mental health, physical health, and overall wellness. While initiatives to enhance the employability of youth are critical within the South African context, our findings of high levels of poor mental health, physical health, and well-being may suggest that young people joining such initiatives are not in an optimal position to utilize these opportunities. With a substantial youth population currently accessing these initiatives each year, YEIs present a rare opportunity to intervene in the futures of previously NEET youth. Integrating programmatic content and support to optimize youth health and well-being while building skills and capacity for economic advancement would have multiple gains for individuals, communities, and the country.

## Data Availability

Data is available from the corresponding author upon reasonable request.
